# Timing of Postpartum Depressive Symptoms

**DOI:** 10.5888/pcd20.230107

**Published:** 2023-11-09

**Authors:** Cheryl L. Robbins, Jean Y. Ko, Denise V. D’Angelo, Beatriz Salvesen von Essen, Connie L. Bish, Charlan D. Kroelinger, Heather D. Tevendale, Lee Warner, Wanda Barfield

**Affiliations:** 1 Division of Reproductive Health, National Center for Chronic Disease Prevention and Health Promotion, Centers for Disease Control and Prevention, Atlanta, Georgia; 2 US Public Health Service Commissioned Corps, Atlanta, Georgia

## Abstract

**Introduction:**

Postpartum depression is a serious public health problem that can adversely impact mother–child interactions. Few studies have examined depressive symptoms in the later (9–10 months) postpartum period.

**Methods:**

We analyzed data from the 2019 Pregnancy Risk Assessment Monitoring System (PRAMS) linked with data from a telephone follow-up survey administered to PRAMS respondents 9 to 10 months postpartum in 7 states (N = 1,954). We estimated the prevalence of postpartum depressive symptoms (PDS) at 9 to 10 months overall and by sociodemographic characteristics, prior depression (before or during pregnancy), PDS at 2 to 6 months, and other mental health characteristics. We used unadjusted prevalence ratios (PRs) to examine associations between those characteristics and PDS at 9 to 10 months. We also examined prevalence and associations with PDS at both time periods.

**Results:**

Prevalence of PDS at 9 to 10 months was 7.2%. Of those with PDS at 9 to 10 months, 57.4% had not reported depressive symptoms at 2 to 6 months. Prevalence of PDS at 9 to 10 months was associated with having Medicaid insurance postpartum (PR = 2.34; *P* = .001), prior depression (PR = 4.03; *P* <.001), and current postpartum anxiety (PR = 3.58; *P* <.001). Prevalence of PDS at both time periods was 3.1%. Of those with PDS at both time periods, 68.5% had prior depression.

**Conclusion:**

Nearly 3 in 5 women with PDS at 9 to 10 months did not report PDS at 2 to 6 months. Screening for depression throughout the first postpartum year can identify women who are not symptomatic early in the postpartum period but later develop symptoms.

**Summary**

**What is already known on this topic?**

Postpartum depression is common and can last long-term. Few studies have examined depressive symptoms late (9–10 months) in the postpartum period.

**What is added by this report?**

We found 7.2% of postpartum women had depressive symptoms at 9 to 10 months after giving birth, 57.4% of whom did not have postpartum depressive symptoms at 2 to 6 months after giving birth. About 3.1% had symptoms of depression at both times.

**What are the implications for public health practice?**

Screening for depression throughout the first postpartum year can identify women who are not symptomatic early postpartum but later develop symptoms.

## Introduction

Postpartum depression is a serious public health problem that can have long-lasting and multigenerational consequences (1). A systematic review of published literature (2005–2016) provides evidence of the negative effects of postpartum depression on maternal health, including relationships, risky behaviors, and quality of sleep; infant cognitive and language development; and mother–child interactions, including bonding and breastfeeding (2). In its most severe form, postpartum depression can result in obtrusive thoughts of and attempts at suicide or infanticide (2). Although suicide has many contributing factors, pregnancy-related mental health deaths (including deaths from suicide, overdose/poisoning related to substance use disorder, and other deaths determined to be related to a mental health condition) account for more than 20% of all pregnancy-related deaths in the US and are the leading cause of preventable pregnancy-related deaths (3).

Postpartum depression is self-reported in approximately 12% to 16% of US women (4). Co-occurring anxiety (5) and substance use (6) are common and can complicate diagnosis and treatment. Population-based studies examining correlates of postpartum depressive symptoms (PDS) have mostly focused on the first 2 to 6 months postpartum (7,8). Professional guidelines on depression during and after pregnancy also focus on screening for depressive symptomology during that period (9,10).

Epidemiologic studies have examined correlates of depressive symptoms later in the postpartum period (11–13), but most studies were based on data from a single state (12,13); one study was based on a nationally representative sample of individuals who gave birth in 2005 (11). Examination of characteristics associated with depression or depressive symptoms in the later postpartum period is important because more than 60% of pregnancy-related deaths due to mental health conditions occur 43 to 365 days postpartum (14). The objectives of this study were to estimate the prevalence and identify the correlates of PDS at 9 to 10 months and estimate the prevalence of PDS at both 2 to 6 months and 9 to 10 months, by using population-based data from 7 states in 2019.

## Methods

We analyzed population-based data from the 2019 Pregnancy Risk Assessment Monitoring System (PRAMS), an ongoing survey (hereinafter referred to as the PRAMS Core Survey) that routinely collects jurisdiction-specific, population-based data on maternal attitudes and experiences before, during, and shortly after pregnancy. In 2019, forty-seven states, the District of Columbia, New York City, and Puerto Rico participated in PRAMS. Each month, a random sample of women with a recent live birth is selected from birth certificate records and is contacted 2 to 6 months after delivery. Participants respond to the PRAMS Core Survey via mail (self-administered) or, among those who do not respond by mail, by telephone (interviewer-administered); the median time from initial outreach to participation was 4 months. The PRAMS Core Survey is implemented through a standardized protocol and questionnaire, described elsewhere (15). Additional details are available from the PRAMS website (www.cdc.gov/prams).

In 2019, PRAMS implemented 2 surveys related to illicit use of opioids and prescription opioid use and misuse, which included a 13-question supplemental module on prescription opioid use and misuse during pregnancy (hereinafter referred to as the Opioid Supplement) and a 58-question telephone follow-up survey 9 to 10 months postpartum (hereinafter referred to as the Call-Back Survey) (16). Thirty-two PRAMS sites and 2 non-PRAMS sites (California and Ohio) implemented the Opioid Supplement (16). The Call-Back Survey was a telephone-only survey that used standardized PRAMS telephone interview procedures for data collection (15) and was fielded in 7 PRAMS sites with high rates of opioid-involved overdose deaths (Kentucky, Louisiana, Massachusetts, Missouri, Pennsylvania, Utah, and West Virginia). Respondents to the PRAMS Core Survey in the 7 jurisdictions were recontacted by telephone to participate in the Call-Back Survey 9 months after the infant’s birth. Respondents had a 31-day window for completing the questionnaire (until 10 months postpartum). All PRAMS respondents in the 7 states were eligible for the Call-Back Survey unless they opted out of being recontacted. Data collection for the Call-Back Survey occurred October 2019 through April 2020.

**Figure 1:**
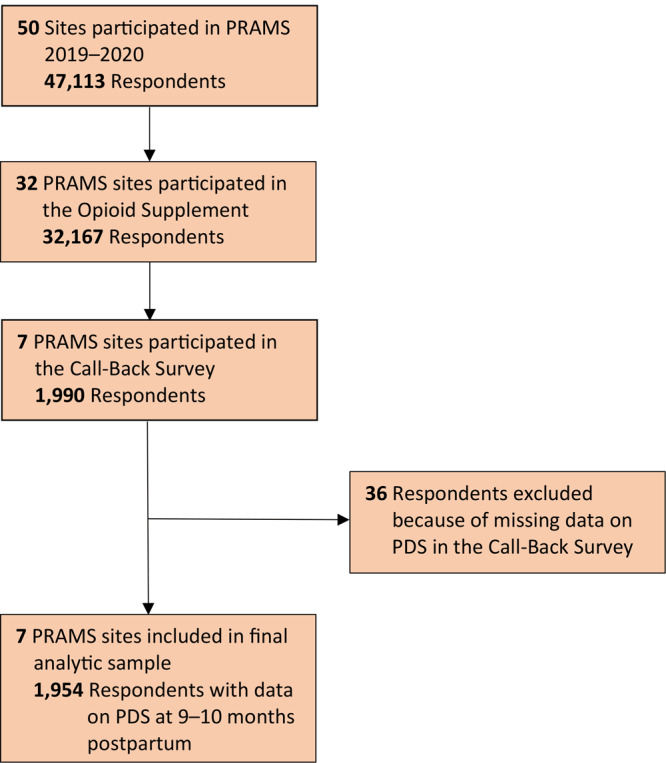
Flow diagram showing how the final analytic sample for the PRAMS Call-Back Survey was derived. Abbreviations: PDS, postpartum depressive symptoms; PRAMS, Pregnancy Risk Assessment Monitoring System.

For this analysis, we linked data from the Call-Back Survey for the 7 sites with the sites’ PRAMS Core Survey data, which includes birth certificate data. The weighted response rates for the 7 participating states ranged from 49% to 73% (median = 58%) for the 2019 PRAMS Core Survey and 45% to 70% (median = 59%) for the Call-Back Survey. The analytic sample included women with data on PDS at 9 to 10 months (Figure 1). The Centers for Disease Control and Prevention (CDC) and each site’s institutional review board reviewed and approved the PRAMS study protocol.

### Outcomes

We assessed self-reported PDS at 9 to 10 months (yes/no) by using a modified version of the 2-item Patient Health Questionnaire (PHQ-2), which aligns with definitions of PDS used in previous surveillance reports (7,8). Two questions assessed frequency of depressive symptoms (always, often, sometimes, rarely, and never) in the past 30 days: 1) “how often have you felt down, depressed, or hopeless?” and 2) “how often have you had little interest or little pleasure in doing things?” We defined PDS at 9 to 10 months as responses of always or often to either question. We also examined PDS at both time points, at 2 to 6 months and at 9 to 10 months, hereinafter referred to as PDS at both time periods.

### Sociodemographic characteristics

We obtained information on sociodemographic characteristics from the linked birth certificate data, including maternal age (≤24, 25–34, ≥35 y), race and ethnicity (Hispanic, non-Hispanic Asian, non-Hispanic Black, non-Hispanic White, and non-Hispanic Other race [included American Indian, multiple races, and other non-White race; no respondents self-identified as Native Hawaiian or Alaska Native]), number of years of education (<12, 12, >12 y), and marital status (married, not married). Information on postpartum health insurance (none, Medicaid, private, other) was self-reported on the Call-Back Survey.

### Perinatal mental health characteristics

Respondents retrospectively self-reported depression 3 months before pregnancy (yes/no) and depression during pregnancy (yes/no) at the time of the PRAMS Core Survey. On the basis of those responses, we created a binary variable to reflect depression before or during the most recent pregnancy (hereinafter referred to as prior depression). Self-reported PDS at 2 to 6 months (yes/no) was assessed at the time of the PRAMS Core Survey by using the same modified version of the PHQ-2 and definition of PDS that were used to define PDS at 9 to 10 months. However, the questions for PDS at 2 to 6 months asked about depressive symptoms since the baby was born (not in the past 30 days). Current anxiety at 9 to 10 months (yes/no) was self-reported at the time of the Call-Back Survey and was assessed by using the prompt “Do you currently have any of the following health conditions [5 items, including anxiety, were listed]?” Respondents who answered yes to the question on anxiety were considered to have anxiety.

### Postpartum substance use

Postpartum substance use was assessed by self-report at the time of the Call-Back Survey (9–10 months). Postpartum smoking (yes/no) was assessed by using the following prompt: “Since your baby was born, have you used cigarettes, e-cigarettes, or any other tobacco products?” We evaluated postpartum alcohol consumption by using responses to the following questions: “Since your baby was born, how many alcoholic drinks did you have in an average week?” and “Since your baby was born, how many times did you drink 4 alcoholic drinks or more in a 2-hour time span?” Heavy drinking or binge drinking (yes/no) was defined as consuming 8 or more alcoholic drinks (ie, wine, wine cooler, beer, liquor, or mixed drinks) per week or 4 or more alcoholic drinks in a 2-hour time span. Postpartum marijuana or cannabidiol use (yes/no) was assessed by using the prompt “Since your baby was born, have you used any of the following medications or drugs for any reason?” The list included 15 types of medications and drugs, including marijuana or hash, cannabidiol, or cannabidiol products.

### Analysis

We calculated the prevalence and 95% CIs of PDS at 9 to 10 months overall and by maternal characteristics (ie, sociodemographic, perinatal mental health, and postpartum substance use). We examined prior depression among those with and without PDS at both time periods. We used unadjusted prevalence ratios (PRs) to examine associations between maternal characteristics and PDS at 9 to 10 months; we considered a *P* value <.05 to be significant. Sample size constraints did not permit multivariate regression modeling. Missing data on maternal characteristics ranged from 0% (age) to 2.0% (self-reported depression during pregnancy) and were excluded from analyses. We conducted all analyses in Stata version 17.0 (StataCorp LLC) and used weighted data to calculate percentages, adjusting for the complex survey design, sampling design, nonresponse, and noncoverage specific to the births within the sampling period of the Call-Back Survey.

## Results

Of 1,954 participants in the analytic sample, approximately one-quarter (24.8%; n = 464) reported prior depression (before or during pregnancy) (Table 1). The prevalence of PDS at 2 to 6 months was 11.9% (n = 269); at 9 to 10 months, 7.2% (n = 161); and at both time periods, 3.1% (n = 75). The prevalence of PDS at 9 to 10 months ranged by PRAMS site from 3.8% (Massachusetts) to 12.4% (West Virginia). Most of the sample was aged 25 years or older (81.2%) and had 12 or more years of education (91.2%) (Table 1). PDS at 9 to 10 months was positively associated with being younger than 24 years (PR = 2.30; 95% CI, 1.42–3.72), non-Hispanic Black (PR = 1.82; 95% CI, 1.10–3.00), and not married (PR = 1.77; 95% CI, 1.13–2.80); having postpartum Medicaid insurance (PR = 2.34; 95% CI, 1.44–3.81); and reporting prior depression (PR = 4.03; 95% CI, 2.53–6.42), current postpartum anxiety (PR = 3.58; 95% CI, 2.20–5.83), postpartum smoking (PR = 2.67; 95% CI, 1.62–4.39), and postpartum marijuana or cannabidiol use (PR = 3.35; 95% CI, 1.81–6.22) (Table 2). PDS at 9 to 10 months was negatively associated with having more than 12 years of education (PR = 0.51; 95% CI, 0.28–0.91).

**Table 1:** Distribution of Maternal Characteristics Overall and by Self-Reported Postpartum Depressive Symptoms at 9 to 10 Months,^a^ Pregnancy Risk Assessment Monitoring System, 2019^b^

Characteristic	Unweighted no. (weighted %)^c^ (N = 1,954)	Postpartum depressive symptoms, weighted % (95% CI)	
		Yes (n = 163)	No (n = 1,791)
Overall	1,954 (100.0)	7.2 (5.7–9.0)	92.8 (91.0–94.3)
**Sociodemographic**			
Age, y^d^			
≤24	375 (18.9)	33.8 (24.2–45.0)	17.7 (15.3–20.4)
25–34	1,176 (61.8)	48.2 (36.7–59.8)	62.8 (59.4–66.1)
≥35	403 (19.4)	18.0 (10.0–30.2)	19.5 (16.9–22.3)
Race and ethnicity^d^			
Hispanic	250 (10.4)	9.4 (5.1–16.6)	10.5 (8.6–12.7)
Non-Hispanic Asian	73 (4.2)	1.5 (0.5–4.8)	4.4 (3.0–6.4)
Non-Hispanic Black	352 (13.9)	22.6 (15.4–32.0)	13.2 (11.1–15.6)
Non-Hispanic White	1,169 (69.2)	62.2 (51.3–72.0)	69.8 (66.5–72.9)
Non-Hispanic Other^e^	69 (2.3)	4.2 (1.7–9.8)	2.2 (1.4–3.3)
Years of education^d^			
<12	188 (8.8)	13.5 (8.2–21.5)	8.4 (6.6–10.7)
12	437 (22.2)	32.9 (22.6–45.0)	21.4 (18.6–24.5)
>12	1,297 (69.0)	53.7 (41.9–65.1)	70.2 (66.8–73.4)
Marital status^d^			
Married	1,200 (64.6)	50.7 (39.2–62.1)	65.7 (62.3–68.9)
Not married	753 (35.4)	49.3 (37.9–60.8)	34.3 (31.1–37.7)
Health insurance at 9 to 10 months^f^			
None	168 (8.2)	5.5 (2.5–11.5)	8.4 (6.6–10.7)
Medicaid	629 (30.0)	50.3 (38.7–61.8)	28.4 (25.3–31.7)
Private	1,098 (59.6)	42.6 (31.4–54.7)	61.0 (57.4–64.2)
Other	41 (2.2)	1.6 (0.5–5.2)	2.2 (1.4–3.6)
**Perinatal mental health**			
Prior depression (before or during pregnancy)^g^			
No	1,447 (75.2)	43.0 (31.7–55.0)	77.7 (74.5–80.6)
Yes	464 (24.8)	57.0 (45.0–68.3)	22.3 (19.4–25.5)
Current postpartum anxiety^f^			
No	1,351 (69.2)	38.6 (27.6–51.0)	71.6 (68.2–74.8)
Yes	601 (30.8)	61.4 (49.1–72.4)	28.4 (25.3–31.8)
**Postpartum substance use (since baby was born)**			
Smoking^f^			
No	1,665 (84.8)	67.6 (55.0–78.1)	86.1 (83.5–88.4)
Yes	288 (15.2)	32.4 (21.9–45.0)	13.9 (11.6–16.5)
Heavy drinking or binge drinking^f^^,^ h			
No	1,755 (91.3)	86.8 (74.9–93.6)	91.7 (89.7–93.3)
Yes	192 (8.7)	13.2 (6.4–25.0)	8.3 (6.7–10.3)
Marijuana use^f^^,^ i			
No	1,837 (94.4)	83.4 (71.2–91.1)	95.3 (93.8–96.4)
Yes	116 (5.6)	16.6 (8.9–28.8)	4.7 (3.6–6.2)

a Postpartum depressive symptoms at 9 to 10 months defined as feeling down, depressed, hopeless, or having little interest or pleasure in doing things usually enjoyed (always or often) in the past 30 days as self-reported at time of Call-Back Survey 9 to 10 months.

b Pregnancy Risk Assessment Monitoring System (PRAMS) Core Survey and Call-Back Survey data from Kentucky, Louisiana, Massachusetts, Montana, Pennsylvania, Utah, and West Virginia; prevalence ratios weighted to adjust for the complex survey design, sampling design, nonresponse, and noncoverage specific to the births within the sampling period of the Call-Back survey.

c Percentages are based on number of respondents for whom we had data; percentages may not sum to 100.0 because of rounding.

d Information obtained from birth certificates.

e Includes American Indian, Other race, and multiple race.

f Based on self-report at time of the Call-Back Survey 9 to 10 months.

g Defined as depression 3 months before pregnancy or during pregnancy; based on self-report at time of PRAMS Core Survey (2 to 6 months postpartum).

h Defined as drinking ≥8 drinks/week or having ≥4 drinks in a 2-hour span since baby was born.

i Defined as using marijuana or hash, cannabidiol, or cannabidiol products since baby was born.

**Table 2:** Associations Between Maternal Characteristics and Self-Reported Postpartum Depressive Symptoms at 9 to 10 Months,^a^ Pregnancy Risk Assessment Monitoring System, 2019^b^

Characteristic	Unadjusted prevalence ratio (95% CI)	*P* value
**Sociodemographic**		
Age, y^c^		
≤24	2.30 (1.42–3.72)	.001
25–34	1 [Reference]	—
≥35	1.19 (0.60–2.38)	.62
Race and ethnicity^c^		
Hispanic	1.01 (0.51–1.98)	.98
Non-Hispanic Asian	0.41 (0.12–1.39)	.15
Non-Hispanic Black	1.82 (1.10–3.00)	.02
Non-Hispanic White	1 [Reference]	—
Non-Hispanic Other^d^	2.01 (0.81–4.97)	.13
Years of education^c^		
<12	1 [Reference]	—
12	0.96 (0.51–1.82)	.91
>12	0.51 (0.28–0.91)	.02
Marital status^c^		
Married	1 [Reference]	—
Not married	1.77 (1.13–2.80)	.01
Health insurance at 9 to 10 months^e^		
None	0.93 (0.40–2.20)	.87
Medicaid	2.34 (1.44–3.81)	.001
Private	1 [Reference]	—
Other	1.06 (0.30–3.69)	.93
**Perinatal mental health**		
Prior depression (before or during pregnancy)^f^		
No	1 [Reference]	—
Yes	4.03 (2.53–6.42)	<.001
Current postpartum anxiety^e^		
No	1 [Reference]	—
Yes	3.58 (2.20–5.83)	<.001
**Postpartum substance use (since baby was born)**		
Smoking^e^		
No	1 [Reference]	—
Yes	2.67 (1.62–4.39)	<.001
Heavy drinking or binge drinking^e^^,^ g		
No	1 [Reference]	—
Yes	1.60 (0.76–3.34)	.21
Marijuana use^e^^,^ h		
No	1 [Reference]	—
Yes	3.35 (1.81–6.22)	<.001

a Postpartum depressive symptoms at 9 to 10 months defined as feeling down, depressed, hopeless, or having little interest or pleasure in doing things usually enjoyed (always or often) in the past 30 days as self-reported at time of Call-Back Survey 9 to 10 months.

b Pregnancy Risk Assessment Monitoring System (PRAMS) Core Survey and Call-Back Survey data from Kentucky, Louisiana, Massachusetts, Montana, Pennsylvania, Utah, and West Virginia; prevalence ratios weighted to adjust for the complex survey design, sampling design, nonresponse, and noncoverage specific to the births within the sampling period of the Call-Back Survey.

c Information obtained from birth certificates.

d Includes American Indian, Other race, and multiple race.

e Based on self-report at time of the Call-Back Survey 9 to 10 months.

f Defined as depression 3 months before pregnancy or during pregnancy; based on self-report at time of PRAMS Core Survey (2 to 6 months postpartum).

g Defined as drinking ≥8 drinks/week or having ≥4 drinks in a 2-hour span since baby was born.

h Defined as using marijuana or hash, cannabidiol, or cannabidiol products since baby was born.

**Figure 2:**
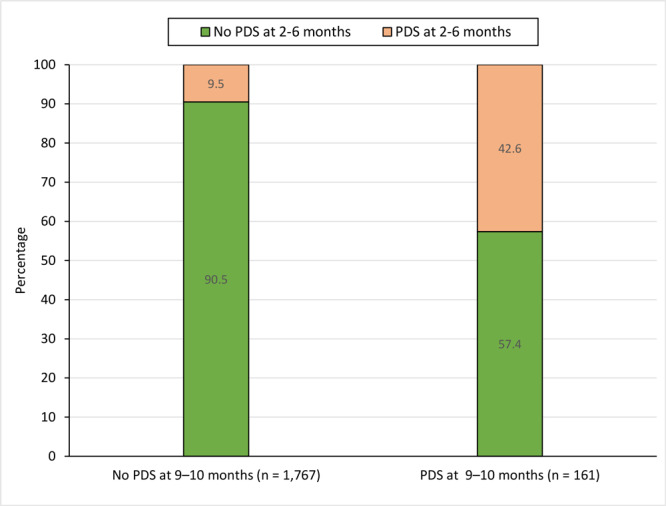
Prevalence of self-reported postpartum depressive symptoms at 2 to 6 months among women with and without postpartum depressive symptoms at 9 to 10 months. Because of missing data for the variable for postpartum depressive symptoms at 2 to 6 months, the number of respondents with and without PDS at 9 to 10 months (n = 1,928) is less than in the full analytic sample (n = 1,954).

Of the 161 respondents with PDS at 9 to 10 months, 86 (57.4%; 95% CI, 45.7%–68.4%) did not report PDS at 2 to 6 months (Figure 2). Of the 75 respondents who reported having PDS at both time periods, 54 (68.5%; 95% CI, 51.2%–81.9%) had prior depression (before or during pregnancy), whereas of the respondents who did not have PDS at both time periods, 403 (23.4%; 95% CI, 20.4%–26.6%) reported prior depression.

## Discussion

In this population-based study of women with live births in 7 US states with high rates of opioid-involved overdose deaths, we found that approximately 1 in 15 women with a recent live birth reported PDS at 9 to 10 months (7.2%) and 11.9% reported PDS at 2 to 6 months. More than half of women with PDS at 9 to 10 months (57.0%) reported having prior depression (before or during pregnancy). Notably, more than half (57.4%) of women with PDS at 9 to 10 months did not report depressive symptoms earlier in the postpartum period (ie, at 2–6 months). We also found that overall, 3.1% of women had PDS at both time periods, and more than two-thirds (68.5%) of those women reported having prior depression.

Our findings on the prevalence of PDS and PDS at both time periods are generally consistent with previously published literature. We used a modified version of the PHQ-2 to assess PDS, and our prevalence estimate of PDS at 9 to 10 months (7.2%) is within the range of other US cohort studies that examined depressive symptoms at 12 months postpartum (range 5%–8%) (12,13). Our finding that 3.1% of women had PDS at both time periods is slightly lower than estimates of persistent perinatal depression from other US studies that followed women for 2 to 3 years postpartum (range 5%–8%) (13,17). Compared with our study, all 3 prior studies were conducted in different locales, during different time periods, and used different depression assessment instruments (12,13,17). For example, Mora and colleagues followed inner-city women living in Pennsylvania from their pregnancy (in 2000–2002) through 2004 and assessed depressive symptomology by using the Center for Epidemiologic Studies Depression Scale (CES-D) (18), with a score of 16 or more suggestive of clinical depression risk (17). Another study, also based in Pennsylvania (12), used data from The First Baby Study (2009–2011) and defined depression as a score of 12 or more on the Edinburgh Postnatal Depression Scale (EPDS) (19). The third study used data from the population-based birth cohort in the Upstate KIDS study in New York State (13), collected data during 2008–2010, and reported mean scores from the EPDS-5 instrument (20). That study reported the prevalence of moderate depressive symptoms by using a cut-off score of 7 or more (13).

The clinical course of perinatal depressive symptom severity and duration varies from person to person, and persistent depressive symptoms can last well beyond the perinatal period (21). One study reported that up to one-quarter of women have elevated depressive symptoms at some point during the 3 years postpartum (13), and another study estimated that one-fifth of mothers continued having depressive symptoms up to 21 years after giving birth (22). Differences in the prevalence and trajectories of PDS have been attributed to the absence of a standardized approach to screening instruments, cutoff scores used for defining depression, and time points of assessment (21).

In line with previous findings, we showed that prior depression (21), comorbid anxiety (5), and substance use (ie, tobacco, marijuana, or cannabidiol use) (6) were associated with PDS. Our estimate of prior depression before or during pregnancy (57.0%) among women with PDS at 9 to 10 months was similar to the estimate from a large screening study at an urban obstetric hospital; the study reported that 60% of women with postpartum depression at 4 to 6 weeks postpartum entered pregnancy with depression or first developed it during pregnancy (23). Consistent with previous findings, we found that rates of postpartum anxiety, smoking, and marijuana or cannabidiol use at 9 to 10 months were higher among women who reported PDS than among those without PDS (5,24).

Our findings highlight the importance of ongoing care and care coordination in the later postpartum period (10,25). Pregnancy-related mental health deaths account for more than 20% of all pregnancy-related deaths in the US and are a leading cause of preventable, pregnancy-related deaths (3). Moreover, from 2008 to 2017, more than 60% of pregnancy-related mental health deaths occurred 43 to 365 days postpartum (14), which speaks to the opportunity for health care providers to ask patients if they have been pregnant in the past year, as promoted in CDC’s Hear Her campaign, to identify urgent maternal warning signs (26).

Clinical guidance for mental health and substance use screening exists for adults in general and specifically for the perinatal period (9,10,27,28). The American College of Obstetricians and Gynecologists’ (ACOG’s) clinical guidelines currently recommend depression, anxiety, and substance use screening at least once during the postpartum period (before 12 weeks postpartum) and transition to follow-up and ongoing care in a primary medical home (10,25). The American Academy of Pediatrics (9) recommends routine screening for maternal postpartum depression during pediatric visits throughout the first 6 months postpartum. The Health Resources and Services Administration’s Screening and Treatment for Maternal Depression and Related Behavioral Disorders program aims to heighten awareness of the importance of screening for maternal mental health conditions, including postpartum anxiety, substance use, and postpartum depression (29). Yet, barriers to postpartum depression screening and treatment remain.

Lack of insurance coverage is a barrier to care for physical and mental health conditions that require ongoing monitoring and care after giving birth (30). Medicaid expansion can increase health insurance coverage to support these important screenings beyond the initial postpartum checkup (25,31). Continuous health insurance coverage through 12 months postpartum can support management of chronic conditions, including depression, after the initial postpartum checkup through coverage of ongoing care (25,31). State Medicaid expansion is associated with improvements in perinatal mental health (32,33). Federal law requires that states extend pregnancy-related Medicaid eligibility from conception through 60 days postpartum for women with household incomes up to the federal minimum standard of 133% of the federal poverty level (34). To address gaps in health insurance coverage during the postpartum period, states also may extend pregnancy-related Medicaid coverage to 12 months postpartum under the American Rescue Plan (35).

Our study contributes to the growing literature that establishes perinatal depressive symptoms can last or even develop well beyond the early postpartum period. It also adds to the evidence of characteristics associated with PDS in the later postpartum period (11–13). By linking data from a one-time call-back survey with routine surveillance data, we demonstrated how existing surveillance systems can be augmented to monitor chronic conditions such as PDS in the later postpartum period and related comorbidities. Similarly, data linkages could capture longitudinal data to examine other predictors of developing depression later in the postpartum period. Future research can examine associations with delivery complications, severe maternal morbidity, and the social determinants of health that underlie structural causes of inequalities in postpartum depression. For example, studies can improve our understanding about how access to educational and employment opportunities and quality health care can affect postpartum depression, particularly PDS, that lasts into the later postpartum period. Other social determinants of health that can be examined in the context of postpartum depression include housing insecurity, food insecurity, neighborhood safety, racial and ethnic discrimination, and other experiences of racism.

### Strengths and limitations

Our study has several strengths. It adds to the limited literature of epidemiologic studies that have examined depressive symptoms later in the postpartum period (11–13) and demonstrates that innovations to augment surveillance systems can be helpful for monitoring chronic conditions such as persistent postpartum depression and related comorbidities. Additionally, the study used data from PRAMS, which had high response rates and small percentages of missing data. However, our findings are subject to limitations. First, depressive symptoms were self-reported on the PHQ-2 and do not necessarily reflect a diagnosis of depression. However, the PHQ-2 has been shown to have high sensitivity (84%) and specificity (79%) for identifying postpartum depression (36). Second, social desirability bias may have resulted in underreporting of stigmatized maternal behaviors and experiences, such as depression and substance use. Third, because of small sample sizes and the relatively small percentage of participants with PDS at both time periods, the data did not permit multivariate regression modeling or comprehensive examination of correlates with that outcome. Small sample sizes also did not permit further examination of PDS by race and ethnicity. Large longitudinal studies with nationally representative data are needed to facilitate multivariable regression analyses that can disentangle the complex relationships between PDS and the correlates examined in this study as well as others, such as social determinants of health. Finally, our results may not be generalizable beyond the study sites. The data represent only women with a recent live birth in the 7 PRAMS sites that implemented the Opioid Supplement and the Call-Back Survey, which used methods during 2019 to oversample counties with high rates of opioid-involved overdose deaths. The findings do not reflect the prevalence of depressive symptoms among women in other parts of the US or women who had other pregnancy outcomes. Additionally, the PRAMS Core Survey and the Call-Back Survey occurred during the early phases of the COVID-19 pandemic, which was associated with increases in psychological distress and depression among the general population (37). Given that timing, estimates of depressive symptoms may be inflated. 

### Conclusions

Our findings, that more than half of women with PDS at 9 to 10 months did not report symptoms earlier in the postpartum period and that 3 in 5 women with PDS at 9 to 10 months had comorbid anxiety symptoms, underscore the importance of screening for depression, anxiety, and substance use throughout the perinatal period (10,28,29).
